# Whole-genome assembly of a hybrid *Trypanosoma cruzi* strain assembled with Nanopore sequencing alone

**DOI:** 10.1093/g3journal/jkae076

**Published:** 2024-04-09

**Authors:** Jill M C Hakim, Sneider A Gutierrez Guarnizo, Edith Málaga Machaca, Robert H Gilman, Monica R Mugnier

**Affiliations:** Department of Molecular Microbiology and Immunology, Johns Hopkins Bloomberg School of Public Health, Baltimore, MD 21205, USA; Department of International Health, Johns Hopkins Bloomberg School of Public Health, Baltimore, MD 21205, USA; Asociación Benéfica PRISMA, Lima 15102, Peru; Infectious Diseases Research Laboratory, Department of Cellular and Molecular Sciences, Universidad Peruana Cayetano Heredia, Lima 15102, Peru; Department of International Health, Johns Hopkins Bloomberg School of Public Health, Baltimore, MD 21205, USA; Department of Molecular Microbiology and Immunology, Johns Hopkins Bloomberg School of Public Health, Baltimore, MD 21205, USA

**Keywords:** *Trypanosoma cruzi*, Chagas, Nanopore, genome assembly

## Abstract

*Trypanosoma cruzi* is the causative agent of Chagas disease, which causes 10,000 deaths per year. Despite the high mortality associated with Chagas, relatively few parasite genomes have been assembled to date, with genome assemblies unavailable even for some commonly used laboratory strains. This is at least partially due to *T. cruzi*'s highly complex and highly repetitive genome, which defies investigation using traditional short-read sequencing methods. In this study, we have generated a high-quality whole-genome assembly of the hybrid Tulahuen strain, a commercially available type VI strain, using long-read Nanopore sequencing without short-read scaffolding. The assembled genome contains 25% repeat regions, 17% variable multigene family members, and 27% transposable elements (TEs) and is of comparable quality with *T. cruzi* genome assemblies that utilized both long- and short-read data. Notably, we find that regions with TEs are significantly enriched for multicopy surface proteins, and that surface proteins are, on average, closer to TEs than to other coding regions. This finding suggests that mobile genetic elements such as transposons may drive recombination within surface protein gene families. This work demonstrates the feasibility of Nanopore sequencing to resolve complex regions of *T. cruzi* genomes, and with these resolved regions, provides support for a possible mechanism for genomic diversification.

## Introduction

*Trypanosoma cruzi* causes Chagas disease, a poorly understood and potentially fatal illness that is estimated to infect 6 million people worldwide. Chagas disease exhibits substantial phenotypic variability: only 30% of infected patients develop symptoms following chronic infection, and these symptoms can involve different organ systems. Moreover, even parasite strains adapted to laboratory culture show phenotypic differences in drug susceptibility, in vitro growth capacity, and experimental infectivity in mice (Brener and Chiari 1965; Bice and Zeledon 1970; Rodriguez *et al*. 2014; Sykes *et al*. 2023). *T. cruzi's* phenotypic diversity is accompanied by a corresponding genomic diversity that is observed across both field isolates and lab-adapted strains (Llewellyn *et al*. 2011; Maiguashca Sánchez *et al*. 2020; Talavera-López *et al*. 2021; Wang *et al*. 2021). While it is not known what factors mediate *T. cruzi's* phenotypic diversity, parasite genetic factors are thought to play a role (Macedo and Pena 1998; Vago *et al*. 2000; Hakim *et al*. 2023).

Understanding which parasite genes contribute to disease and phenotypic variability first requires a knowledge of *T. cruzi* genomic diversity. In the absence of full-genome sequences, *T. cruzi* isolates are usually categorized into 6 genotypes termed discrete typing units (DTUs), based on the results of several PCR and gel electrophoresis steps. However, these genotypes insufficiently describe the full gamut of parasite diversity and are not strongly associated with any clinical phenotype (Zingales *et al*. 2012; Messenger *et al*. 2015).

Unfortunately, there are few high-quality whole genomes available for *T. cruzi*. This is in part attributed to the fact that the basic features of the parasite's genome are difficult to study due to the genome's repetitiveness, variability in genome sizes, number of chromosomes, spontaneous aneuploidies and polyploidies, and lack of synteny between strains. Moreover, 2 parasite genotypes (DTUs V and VI) are a result of hybridization between other parasite genotypes, yielding highly heterozygous genomes that are even more difficult to resolve (Sturm *et al*. 2003). Additionally, what accounts for most of the genomic diversity between strains are 6 highly diverse and multicopy gene families, referred to as multigene families (MGFs) (Maiguashca Sánchez *et al*. 2020; Wang *et al*. 2021), which are distributed throughout the genome and thought to play important roles in immune evasion and parasite pathogenicity (Durante *et al*. 2017; de Fonseca *et al*. 2019; Herreros-Cabello *et al*. 2020). These families further complicate genome assembly, contributing to the repetitiveness of the genome and rendering them difficult to individually resolve or place within their genomic contexts.

Long-read sequencing has increased in accuracy and accessibility in recent years. Recent *T. cruzi* genome assemblies have demonstrated the quality improvement of genomes assembled with long reads, but only when additional Illumina sequencing was used for polishing (Berná *et al*. 2018; Callejas-Hernández *et al*. 2018; Díaz-Viraqué *et al*. 2019; Wang *et al*. 2021). The long-read approach allows the circumvention of several challenges posed by the *T. cruzi* genome: resolution of repetitive regions, accurate placement of diverse gene family members, as well as full resolution of transposable elements (TEs), which are difficult to study in most organisms. Like the MGFs, TEs are difficult to study using short reads alone. They are highly repetitive, can be multiple kilobases long, and can be distributed anywhere in the genome. *T. cruzi* has the highest proportion of TEs in its genome of any pathogenic kinetoplastid parasite, and TEs are hypothesized to play roles in both the unique transcriptional control of the parasite and the facilitation of genomic rearrangements (Bringaud *et al*. 2006, 2008; Souza *et al*. 2007; Thomas *et al*. 2010; Macías *et al*. 2018; Pita *et al*. 2019; Gómez *et al*. 2021).

We sought to develop a scalable pipeline for generating *T. cruzi* genomes to fully resolve these multigene families and TEs. We chose to develop this pipeline using the Tulahuen strain, which is commercially available from the American Type Culture Collection (ATCC) and has been used in numerous experimental studies, especially drug susceptibility studies, due to its endogenously expressed LacZ reporter (Buckner *et al*. 1996; Mercado *et al*. 2019). Moreover, this parasite strain is a type VI DTU, 1 of the 2 hybrid DTUs, which are the most common genotypes in the southern cone of the Americas, where the burden of Chagas disease is high. Because many people are infected with these hybrid strains, it is of clinical importance to understand their genomes. Despite its relative importance, up to now there has been no publicly available genome for the Tulahuen strain, potentially limiting its experimental utility.

In this study, we have used Oxford Nanopore Technology (ONT) long-read sequencing, without supplementation with short Illumina reads, to generate a whole genome of the Tulahuen LacZ clone C4 strain. We find a high proportion of heterozygosity in the Tulahuen genome and annotate the genome for multigene family members and TEs. Using the newly annotated genome, we find that the distance between a TE and an open reading frame (ORF) is bimodally distributed, and multigene family members fall exclusively within the histogram peak closest to TEs. This observation holds across multiple *T. cruzi* genomes, as well as in *Trypanosoma brucei*, suggesting that this genome organization is common to trypanosomes. This new genome provides critical information for basic biological research and raises interesting questions about the mechanisms driving virulence factor diversification.

## Methods

### Parasite culture

*T. cruzi* epimastigotes (Tulahuen LacZ clone C4, BEI resources: NR-18959, DTU: VI) were grown in liver infusion tryptose medium supplemented with 10% heat-inactivated fetal bovine serum, 100 U/mL penicillin (Sigma-Aldrich, P3032), and 100 μg/mL streptomycin (Sigma-Aldrich, S9137). Parasites were seeded at a density of 1 × 10^6 ^cells/mL and harvested during the early stationary phase for DNA extraction.

Parasite DNA was extracted using the NEB HMW kit (NEB, T3050S), then left at 4°C for at least 2 weeks prior to sequencing to relax tightly coiled DNA prior to sequencing. Using ligation sequencing kits (112 and 114, catalog numbers SQK-LSK112 and SQK-LSK114), ONT libraries were prepared and sequenced on 2 flow cells (9.4.1 and 10.4.1, respectively). The cumulative output from these 2 runs was 3.6 Gb and the N50 was 10 kb. Raw Pod5 files were base-called by using the Guppy super accuracy model, and duplex reads were called from data produced using the 10.4.1 flow cell and then integrated into the overall data.

### Genome sequencing and assembly

A kmer histogram for 21-mers was generated with Jellyfish, and then genome heterozygosity was estimated by using GenomeScope (Marçais and Kingsford 2011; Ranallo-Benavidez *et al*. 2020). Fastq data from 9.4.1 and 10.4.1 were pooled and blasted against a database of known kDNA sequences. Raw reads that match kDNA were removed using seqkit, and the remaining reads were assembled using Nextdenovo and then polished once using Nextpolish using default parameters for both softwares; specific parameters are included in the github repository as config files (Hu *et al*. 2020, 2023). Read coverage was assessed by mapping the raw reads to the assembly with minimap2, and the average coverage for each contig was determined by using samtools cov (Li 2018). A search for Benchmarking Single-Copy Orthologs (BUSCO) genes was performed using the euglenozoan v10 database (Manni *et al*. 2021). Genomes used for BUSCO comparisons were retrieved from TriTrypDB or NCBI (El-Sayed *et al*. 2005; Franzén *et al*. 2011; Reis-Cunha *et al*. 2015; Baptista *et al*. 2018; Berná *et al*. 2018; Bradwell *et al*. 2018; Callejas-Hernández *et al*. 2018; Díaz-Viraqué *et al*. 2019; DeCuir *et al*. 2021; Wang *et al*. 2021).

### Genome annotation

Coding sequences were annotated in 2 steps: First, genome annotations from the assembly of the Brazil A4 genome were mapped onto the new Tulahuen assembly using LiftOff (Shumate and Salzberg 2021). Second, additional de novo gene calls were produced using AUGUSTUS trained on the Cl Brenner genome, which has fewer annotated genes than the Brazil strain, but is manually curated (Stanke and Morgenstern 2005). These results were combined with GFFread –merge function (Pertea and Pertea 2020).

Multigene family members were further annotated using a method adapted from Wang *et al*. (2021). Briefly, a blast search against a database of known multigene family members was performed against the previously described coding regions with a % identity cutoff of 85 and a length cutoff of 150 bp. If a genomic region matched multiple MGFs, the annotation was assumed to be nonspecific to either family and was removed. To identify pseudogenes encoding for multigene family members, the search was performed against the entire assembly, and the genes that were found outside the coding regions were defined as pseudogenes.

TE annotation was performed using the Extensive De Novo Transposon Annotator with the sensitive parameter (Ou *et al*. 2019).

All downstream analyses and figures were generated in R. All scripts used to generate the assembly, annotations, and analyses are available at https://github.com/mugnierlab/Hakim2023. Raw data and the final assembly have been deposited at BioProject PRJNA1011856. All genome annotations are available at the linked github.

## Results

### Nanopore sequencing alone produces a complete whole genome

**Fig. 1.**
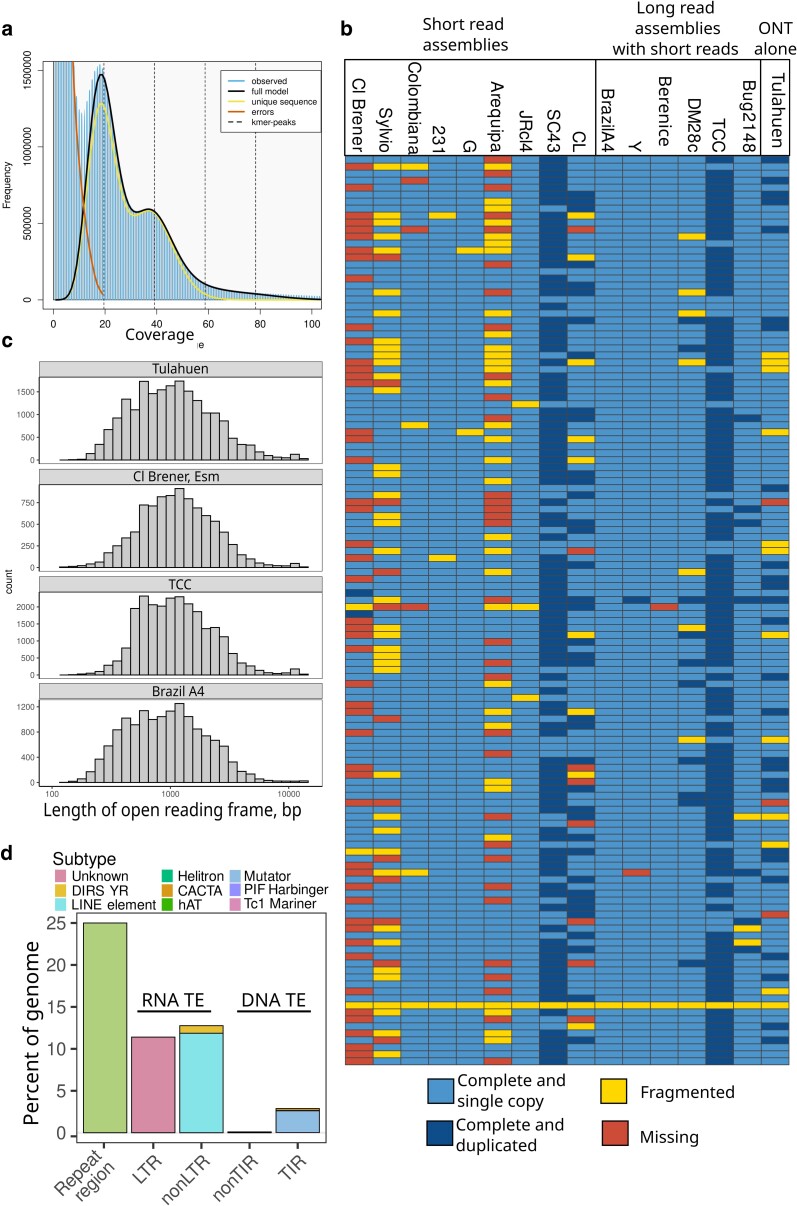
A whole-genome assembly of a reference hybrid strain. a) A genomescope 21-mer histogram on raw nanopore reads. b) BUSCOs for the newly assembled Tulahuen genome, as well as other *T. cruzi* genomes assembled with different data types. The columns indicate strains, and the rows indicate BUSCOs. c) A histogram of lengths for ORFs in Cl Brener, Brazil A4, and Tulahuen genomes. d) A summary of TE classes found in the Tulahuen genome. CACTA, CACTA terminal inverted repeat, element; DIRS_YR, tyrosine recombinase TE; LINE, long interspersed nuclear element; LTR, long terminal repeat; TIR, terminal inverted repeat.

DNA from Tulahuen epimastigotes was extracted and sequenced using ONT R9.4.1 and R10.4.1 flow cells. Data from both runs were combined, and genome heterozygosity was estimated using GenomeScope. The genome is highly heterozygous, which is evident by a larger first peak in the kmer frequency distribution histogram, with an alternate allele frequency of 3.04% (Fig. 1a). This high proportion of heterozygosity agrees with the ancestral history of this parasite strain: as a type VI DTU, Tulahuen is a hybrid of 2 other parasite genetic types (de Freitas *et al*. 2006). GenomeScope reports a haploid genome size of 48 Mb, which is within the range of haploid genome sizes of other recently published genomes (45 and 53 Mb; Supplementary Table 1). After assessing heterozygosity, we assembled the genome using NextDenovo, which resulted in a partially phased genome of 48.6 Mb in 75 contigs, with an assembly N50 of 872 kb and a mean coverage of 57×. Of the 75 contigs, 12 had telomeric repeats, and 1 contig had telomeric repeats at both ends, indicating 1 full chromosomal assembly. When measuring the average coverage of each contig, we found a population of contigs with ∼25× coverage and a population of contigs with ∼50× coverage, indicating that the prior set is likely more heterozygous, while the higher-coverage set likely has more regions of homozygosity, and these regions of homozygosity resulted in collapsed contigs (Supplementary Fig. 1). Contigs with coverage higher than 50× likely represent collapsed contigs that have more heterozygosity at sites along the contig or unresolved repeats.

We then used BUSCO scores to assess assembly completeness based on the presence of conserved orthologous genes shared in the euglenozoan phylum. We found that the Tulahuen assembly had a BUSCO completeness of 88.7%, which was slightly lower than that of recent assemblies using both short and long reads, although higher than that of most assemblies using short reads alone, including the reference CL Brener assembly (Fig. 1b). Notably, we found that 1 BUSCO gene, which encodes for a UV excision repair RAD23-like protein, was reported as fragmented in every *T. cruzi* assembly assessed here, indicating that the gene is likely divergent from orthologs in other euglenozoan organisms.

It is important to note that BUSCO completeness, although a useful benchmarking tool for the recovery of conserved, nonrepetitive regions of the genome, may fail to accurately assess the resolution of more complex, repetitive regions of a genome, especially if the single-copy genes are less likely to occur within repetitive regions. For example, our attempt at assembling this genome using Flye produced a genome that was 96% BUSCO complete, but only 26 Mb long, suggesting that the conserved regions were well resolved in this assembly, but that much of the genome made up by repetitive regions, especially diverse MGF members and TEs, were lost. To more fully assess the accuracy of a *T. cruzi* genome assembly, additional analyses may be beneficial, especially ones tailored to a specific assembly method's known systematic errors, such as ONT's known issues in homopolymer resolution.

To evaluate systematic errors of the Nanopore-only assembly, we took advantage of a NEO:LacZ construct cloned into the nuclear genome of the Tulahuen strain available from ATCC (Buckner *et al*. 1996). We found LacZ tandemly expressed 10 times within the insertion locus: both the LacZ and the Neo sequences shared 99% identity with the insertion construct (3,049/3,050 and 790/792, respectively). However, all detected tandem LacZ and Neo constructs were identical to each other in this assembly, suggesting that the differences between the recovered sequences and the published constructs are likely true SNPs and not a result of sequencing or assembly error. Importantly, we found no indels in this region, which is a known systematic error of Nanopore sequencing that typically requires short-read supplementation to correct. Our ability to resolve this known sequence without indels is likely a result of the sequencing data from new 10.4.1 chemistry.

To further assess whether our assembly was affected by a systematic error that led to indels in the assembly, we compared the average ORF length between this assembly and the reference genome, Cl Brener, and 2 other high-quality long-read assemblies, Brazil A4 and TCC. We chose Brazil A4 because of its high BUSCO completeness and TCC because it is also a hybrid strain. Indels in low-complexity regions are problematic when estimating ORFs, as indels will cause frame shifts across the whole contig and result in erroneously short predicted ORFs. We found that the distribution of ORF lengths was comparable for each genome (Fig. 1c). These data further highlight the accuracy of the 10.4.1 chemistry.

### TEs are physically closer to multigene family members than to other ORFs

Following assembly, we annotated the genome for repetitive regions that are generally difficult to resolve during assembly, specifically multigene family members and TEs. We found that a large proportion of the genome was made up of these elements, which again is in agreement with previous work. Twenty-five percent were simple repeats, 27% were TEs, and 22.7% were MGF members. We found many RNA TEs, the most abundant of which were long interspersed nuclear elements and long terminal repeat retrotransposons (Fig. 1d). The automated software also reported the presence of DNA transposons, which have not previously been reported in *T. cruzi* genomes. However, the authors of the TE annotation software used here benchmarked their tool against high-quality TE annotations and found high false discovery rates for terminal inverted repeat transposons (between 36 and 20% depending on the organism). Additional manual curation and experimentation will be required to confirm whether these sequences are true DNA transposons.

**Fig. 2.**
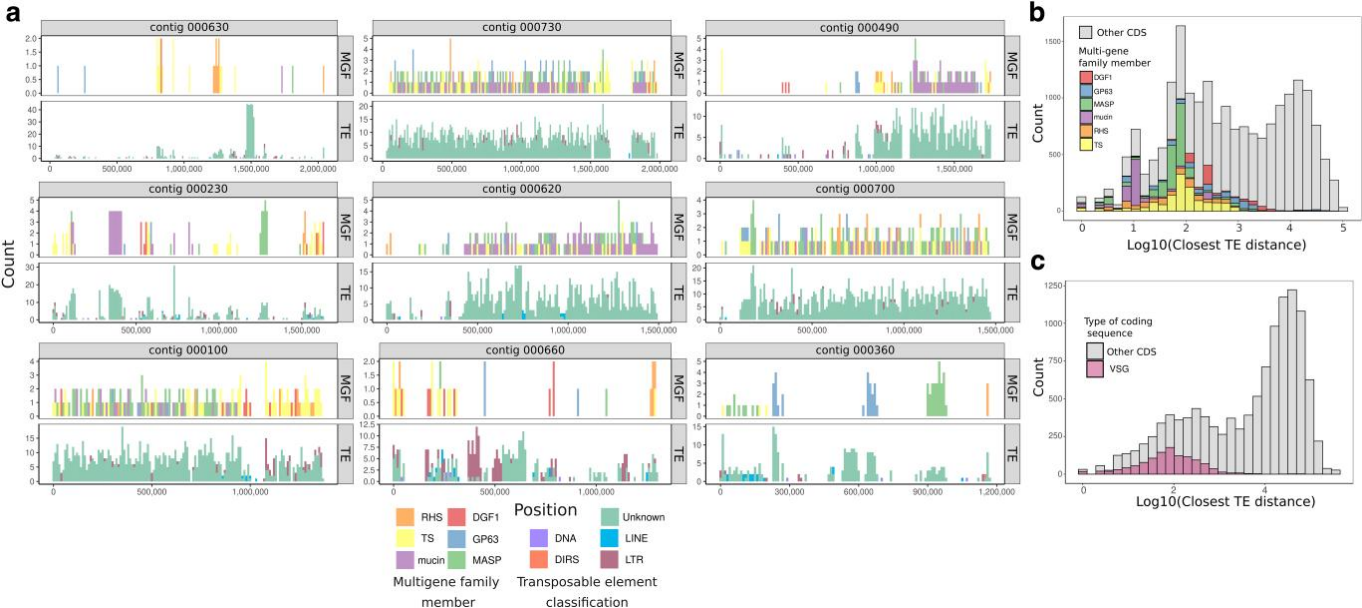
Transposable elements are associated with multigene family members. a) A chromosomal distribution of MGFs (top facet) and TEs (bottom facet) in 10 kb windows across the 9 largest contigs of the assembly. A histogram of the distance between the start and the end of a TE to the start and end of any coding sequence (CDS) in the (b) Tulahuen *T. cruzi* genome and the (c) 427 *T. brucei* genome. In a), multigene family members are in stacked colored bars. All other CDSes are in gray. In b), genes annotated as variable surface glycoproteins are indicated, and all other CDSes are in gray.

To investigate the hypothesis that TEs contribute to the diversification of multigene families, we evaluated the distribution of TEs in relation to ORFs. If TEs were likely to facilitate the movement of multigene family members to different locations in the genome, we would expect the 2 types of genes to be physically close to each other. We found that MGF members and TEs were distributed fairly uniformly throughout the genome, and often occurred in the same 10 kb windows (Fig. 2a). Additionally, we found that the log distance between coding sequences and TEs was bimodally distributed, with multigene family members found closer to TEs than to other ORFs (Fig. 2b). This same pattern was observed in other assemblies such as the high-quality Brazil A4 (Pacbio and Hi-C) and Bernice (Nanopore and Illumina) genomes, indicating that this is unlikely to be an assembly artifact and rather represents a common aspect of *T. cruzi* biology (Supplementary Fig. 2). Moreover, an additional analysis of *T. brucei* TEs and the highly variable, actively diversifying variant surface glycoprotein (VSG) genes showed a similar pattern (Fig. 2c;  So *et al*. 2022). In the *T. brucei* genome, while VSG genes were unilaterally closer to TEs, there were other coding sequences that also seemed enriched around TEs; a closer look at these other coding sequences in this genome revealed many genes involved in TE biology, such as reverse transcriptase and RNAse H (Supplementary Table 2). This observation further supports the hypothesis that TE-mediated diversification may be evolutionarily conserved in trypanosomes.

## Discussion

We have produced a full genome for the hybrid *T. cruzi* Tulahuen strain, which is frequently used in biomedical research. This is the first *T. cruzi* genome to be assembled with ONT long reads alone, without supplementation with other technologies, and the quality of this genome is comparable with that of other high-quality genomes. Despite the complexity of this genome, it was assembled with relatively few reads (a total of 3.6 Gb). Low startup costs of Nanopore technology compared with Illumina or Pacbio, as well as the relatively low-sequencing depth required, suggest that Nanopore sequencing may prove to be an excellent tool for generating whole genomes from a large number of strains, especially in low-resource settings. The main systematic error, indels within homopolymers, seems to have a minimal effect on the predicted ORF lengths in this assembly; this is likely due to the new R10.4.1 sequencing technology, which in independent assessments shows improved resolution for these challenging regions (Sereika *et al*. 2022).

Using the long-read assembly, we were able to annotate multigene family members and TEs and describe their relationship to each other within a linear genome sequence. TEs in *T. cruzi* are known to frequently cluster together within the genome (Olivares *et al*. 2000). Novel to this study is our observation that there seems to exist a genomic compartment containing coding regions that are isolated from TEs; this could point to negative selection keeping potentially deleterious insertions and rearrangements away from important and conserved genome compartments.

Unlike other highly variable gene families, such as VSG genes in *T. brucei* and var genes in *Plasmodium falciparum*, multigene families in *T. cruzi* are not primarily localized to the subtelomere, which is often a hotspot for diversification driven by mitotic recombination. The dispersed nature of *T. cruzi*'s variable genes suggests an alternative, or additional, mechanism for diversification. The data presented here and elsewhere suggest a possible role for TEs in this putative mechanism. There is further evidence of TE's association with MGFs: TcTREZO, a site-specific retrotransposon, shows frequent insertion into MASP genes, and is generally found in nonsyntenic regions of the *T. cruzi* genome containing MGFs (Souza *et al*. 2007). Further genomic analysis of actively diversifying strains isolated from field samples is required to fully characterize the diversity within these multigene families, and additional experimental strategies will help uncover the specific mechanisms underlying any TE-mediated diversification processes.

Very few whole genomes of *T. cruzi* exist, despite the parasite's substantial genetic diversity and the putative role parasite genetics may play in disease heterogeneity. Further, even fewer genomes of hybrid *T. cruzi* genomes exist. These strains are more genomically complex, but critical to understanding parasite diversity, as the majority of parasites in the southern cone of the Americas are hybrid strains. This work demonstrates the feasibility of using Nanopore sequencing alone, at a relatively low-sequencing depth, to study important genomic features such as multigene family members and TEs in a hybrid strain. These new and improving tools will allow us to generate more whole-genome data for this enigmatic and genetically unstable parasite.

## Supplementary Material

jkae076_Supplementary_Data

## Data Availability

All downstream analysis and figures were generated in R. All scripts used to generate the assembly, annotations, and analyses are available at https://github.com/mugnierlab/Hakim2023. Raw data and the final assembly have been deposited at BioProject PRJNA1011856. Supplemental material available at G3 online.
