# Supplementary material for: Whole-genome assembly of a hybrid Trypanosoma cruzi strain assembled with Nanopore sequencing alone
Source: G3 (Bethesda). 2024 Apr 9;14(6):jkae076. doi: 10.1093/g3journal/jkae076 (PMC11152063; doi:10.1093/g3journal/jkae076)
Supplement: jkae076_Supplementary_Data [file jkae076_supplementary_data.zip › Supplemental_Figure_Legends_G3-2024-404973.docx]

Supplemental figure 1:

Histogram of average read depth of raw reads to each assembled contig. A) Histogram of all contigs in the assembly and B) histogram with all contigs except Ctg130 is censored. Ctg130 is an outlier with unusually high read depth in a small region of the sub-telomoeres.

Supplemental figure 2:

Histogram of the distance between the start or end of a transposable element to the start or end of any coding sequence (CDS) in the (A) Brazil *T. cruzi* genome and the (B) Berenice *T. cruzi* genome. Multi-gene family members are in stacked colored bars. All other CDSes are grey.

*Trypanosoma cruzi* is the causative agent of Chagas disease, and has a complex genome that makes it difficult to study large families of repetitive genes important for parasite pathogensis. Using Nanopore sequencing alone, we produce a new *T. cruzi* genome and annotate these large gene families as well as transposable elements. We find that these large gene families are closer to transposble elements than other genes, suggesting that transposable elements are involved in the diversification of these large gene families.
