# Supplementary figures and images for: Whole-genome assembly of a hybrid Trypanosoma cruzi strain assembled with Nanopore sequencing alone
Source: G3 (Bethesda). 2024 Apr 9;14(6):jkae076. doi: 10.1093/g3journal/jkae076 (PMC11152063; doi:10.1093/g3journal/jkae076)

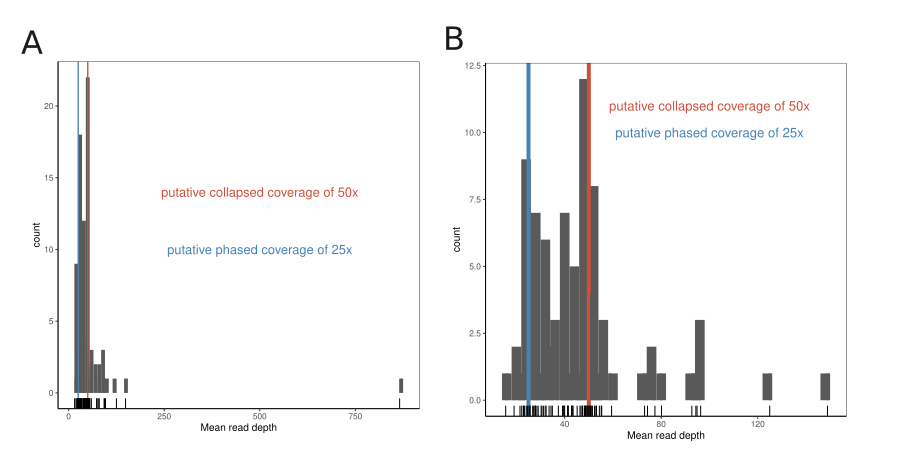

Supplement: jkae076_Supplementary_Data [file jkae076_supplementary_data.zip › Supplemental_Figure_1_G3-2024-404973.png]

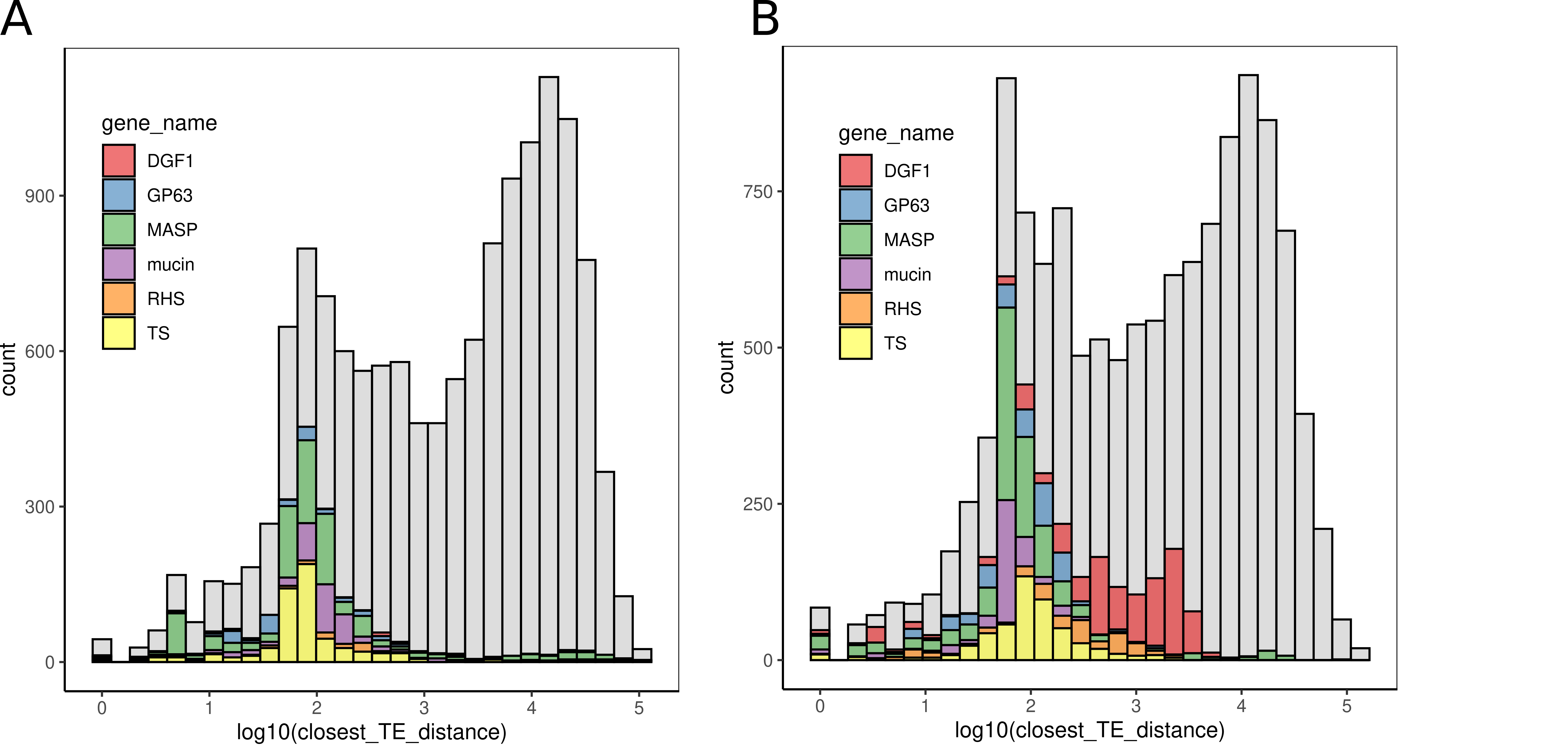

Supplement: jkae076_Supplementary_Data [file jkae076_supplementary_data.zip › Supplemental_Figure_2_G3-2024-404973.png]
